# Protection of perinatal mental health during the war in Ukraine

**DOI:** 10.1016/j.lanepe.2022.100362

**Published:** 2022-03-16

**Authors:** Chiara Sacchi

**Affiliations:** Department of Developmental and Social Psychology, University of Padova, via Venezia 8, Padova, Italy

The dreadful days of war in Ukraine are producing what has already been described as the fastest mass migration to Europe in at least three decades. According to UNHCR, during the first week of Russia's invasion, 1 million people fled Ukraine for neighboring countries, and most of the migrants crossing Poland's borders are women with children.^1^ Refugee women giving birth in desperate conditions are unbearable documented images, and UNFPA estimates that 80,000 women will give birth in the next three months in Ukraine with severely compromised maternal health conditions.^2^

For war-trauma victims, childbirth can be a deeply distressing experience, triggering trauma-response symptoms and producing a dangerous ripple effect for mother-infant health.^3^ To add to this burden, the alarming implications of this humanitarian crisis and war-enforced migration for the perinatal mental health do not stop with immediate exposure. Pregnancy and post-partum represent influential nodes for programming effects on the health of the women, the infants, and their relationship.^4^ While resisting diverse traumatic experiences (e.g., physical harm, loss, extreme fear, food shortages, hibernation, unassisted childbirth), neurobiological and emotional-behavioral interactions continuously occur between a woman and her unborn/newborn baby, exposing both dyad members to the risks of psychophysiological transmission of traumatic stress, which pave for them a dangerous cascade of lasting health problems.

It is therefore of highest priority to minimize the likelihood of re-traumatization of refugee pregnant women and new mothers and safeguard their and their infants’ health by mitigating stress and promoting resilience through the provision of trauma-informed and migration-informed perinatal mental health care. Implications for practice might include: ensuring calm, predictable and safe maternity care environment, facilitating refugee women's experience of medical practices in the host country; providing training on trauma and migration to maternity care staff members; enhancing individualized care: pregnancy and war are very private experiences and disclosure of difficult events should be handled with great sensitivity and trustworthiness; promoting accessible perinatal mental health services, that support women's emotional well-being, empower maternity and prenatal bonding and prevent from traumatic stress risks.

Humanitarian emergencies have been seen as opportunities for mental health reforms; when it comes to perinatal mental health policies a mapping across Europe reveals uneven realities.^5^ Protecting and monitoring perinatal mental health in the response to the war crisis in Ukraine is the paramount opportunity for European countries to reduce the health costs associated with poor perinatal health and prevent from an intergenerational transmission of adversity and trauma.

## Declaration of interests

I declare no competing interests.
